# Study on the freeze–thaw effect on concrete arch dam using an improved response surface method

**DOI:** 10.1038/s41598-023-46544-8

**Published:** 2023-11-06

**Authors:** Qian Cai, Miaofan Yang, Jiangui Yang

**Affiliations:** 1 Nanjing Hydraulic Research Institute, Nanjing, 210029 China; 2 Hohai University, Nanjing, 210024 China; 3 Management Office of Nanjing Sancha River Estuary Sluice, Nanjing, 210036 China

**Keywords:** Civil engineering, Mechanical properties

## Abstract

The damming materials will be deteriorated during the long-term service of concrete arch dam by weathering, seepage dissolution, freeze–thaw and other factors which will put dams at potential risk. However, there are few researches on the reliability assessment of arch dams considering the effect of material deterioration. In this paper, a prediction model of concrete mechanical properties under freeze–thaw cycles is established using regression analysis. Considering various uncertain factors, a time-varying structural reliability analysis model is established using finite element equivalent stress method and an optimized response surface method with improved sampling strategy and convergence criterion. A case study is later carried out. The results show that, at the early stage of freeze–thaw, the structural failure probability will increase steadily in a small range. However, as the tensile strength of concrete continues to decrease under long-time freeze–thaw action, the reliability of the arch dam structure will suddenly fall off a cliff. The proposed deterioration model and reliability analysis probability model are reasonable and practical and could be serve as a technical support for similar engineering projects.

## Introduction

Arch dam is a very economical high order statically indeterminate shell structure which could make full use of arch effect to maximize load bearing. China has a large number of arch dams, accounting for 40% of the world^1^. Most of them were built between the 1950s and 1970s which are close to the standard service life. With the increase of service time, the damming materials will be deteriorated by weathering, seepage dissolution, freeze–thaw and other factors which will put dams at potential risk.

At present, the deterioration of concrete materials under freeze–thaw conditions has been studied intensively^2–4^. Trubic^5^ provided an analysis of the air voids parameters in fresh concrete and the proving of the concrete resistance to the freezing and thawing effects already in fresh concrete. Hong^6^ studied the degradation law of mechanical properties of concrete subjected to freeze–thaw cycles. When damage degree is less than 40%, the compressive strength of freeze–thaw damaged concrete is not lower than 70% of its original strength. Paul^7^ presented an assessment of the freeze–thaw resistance of preformed foam cellular concrete. The results showed that compressive strength, depth of initial penetration, absorption and absorption rate are the important variables in producing cellular concrete that is resistant to cycles of freezing and thawing. Density and permeability were shown not to be significant variables. However, there are few researches on the reliability of arch dams considering the effect of material deterioration. David et al. conducted an investigation of a 40-years old concrete arch dam and studied the concrete deterioration characteristics through coring test, sonic test and petrographic examination^8^. Many other concrete structures in long-term service have also been studied^9–11^. The concrete deterioration characteristics are focused in the above researches. Relevant material deterioration models and structural safety and reliability evaluation models of arch dams are not studied and established. Wang et al. introduced a concrete degradation model with aging because of chemo-mechanical damage and studied the seismic response of an arch dam with aging effects^12^. Pan et al. proposed a unified approach for long-term behavior and seismic response analysis of AAR (alkali-aggregate reaction) -affected concrete dams by combining AAR kinetics, effects of creep and plastic-damage model in the finite element method^13^. Joshi et al. studied the effect of alkali silica reaction on the deflection of an arch dam^14^. The currently proposed degradation models did not take into account many uncertain factors, such as external load uncertainty, structural mechanical uncertainty and so on. The structural reliability research is relatively scarce. Yilmaz et al. proposed a stochastic deterioration modelling approach to evaluate the performance deterioration of corroded concrete structures caused by reinforcement corrosion^15^. However, it does not reflect the effect of concrete deterioration on arch dam. In general, the reliability of the arch dam structure under freeze–thaw effect, considering various uncertainty factors, still needs further study.

In this paper, a freezing–thawing deterioration material model of concrete is proposed through regression analysis. The time-varying strength and reliability of an arch dam under freezing–thawing action are analyzed by using a finite element equivalent stress method and response surface method. The research results provide a method and basis for the safety evaluation of arch dam structure considering material deterioration. Other material deterioration progress, such as weathering, seepage dissolution, et al., could also be similarly analyzed by the proposed method.

## Deterioration model of concrete under freeze–thaw effect

freeze–thaw action is generally considered to be the internal microstructure changing process of cement mortar due to repeated freezing and melting of internal moisture. The freeze–thaw action mainly affects the strength and elastic modulus of concrete. Under freeze–thaw effect, the dynamic elastic modulus of concrete *E*_*D*_ gradually decreases with the increase of freeze–thaw times, and the change rule is basically the same. In order to study the time-varying reliability process of arch dam under static condition, the dynamic elastic modulus *E*_*D*_ needs to be transformed into static elastic modulus *E*_*S*_. According to “Standard for seismic design of hydraulic structures” (GB 51247-2018)^16^, under seismic conditions, the dynamic elastic modulus of concrete increases by 50% on the basis of static elastic modulus. It could be assumed that the dynamic elastic modulus of concrete under freeze–thaw action remains the same as 1.5 times of static elastic modulus, namely:

1 $$E_{D} = 1.5E_{S}$$

**Figure 1:**
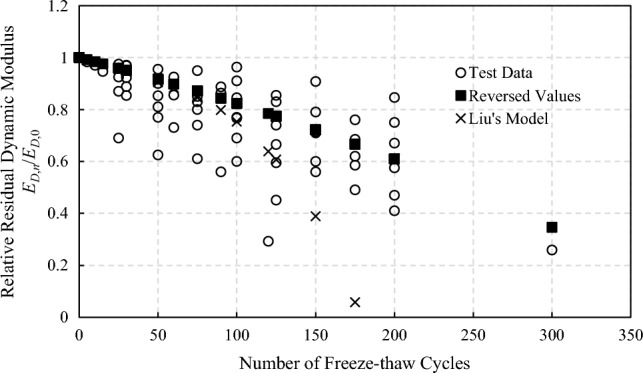
Reversed dynamic elastic modulus of concrete under freeze–thaw action.

Based on the exponential fitting model regression analysis of the dynamic elastic modulus test data in relevant literatures^2, 17–23^, the test data of plain concrete are extracted. And the static elastic modulus of concrete under freeze–thaw action could be reverse analyzed and obatined which is shown in Fig. 1:

2 $$E_{S,n} { = }\frac{2}{3}E_{D,n} { = }\frac{2}{3}{(1}{\text{.7948}} - 0.{7948} \times e^{n/500} )E_{D,0} { = (1}{\text{.7948}} - 0.{7948} \times e^{n/500} )E_{S,0}$$

where *E*_*S*,*0*_,* E*_*D*,*0*_ are the initial static and dynamic elastic modulus of concrete without freeze–thaw action, respectively; *E*_*S*,*n*_, *E*_*D*,*n*_ are the static and dynamic elastic modulus of concrete after *n* times of freeze–thaw action.

Based on a set of freeze–thaw cycle tests, Liu^22^ also proposed a deterioration model which is shown in Eq. (3) and Fig. 1.

3 $$E_{D,n} { = }\frac{{100 \cdot e^{{ - \frac{14526.0423n}{{(202.51^{2} - n^{2} )^{1.5} }}}} - {1}{\text{.13}} \cdot \frac{\pi }{2} - {\text{arctan[0}}{.23} \cdot (n - 102.32)]}}{100}E_{D,0}$$

It could be noted that within the first 75 freeze–thaw cycles, the results of the model proposed in this paper are not much different from Liu's model. However, the failure of Liu's concrete strength test after 200 cycles caused the regression model to decline rapidly after 75 cycles. As more experimental data are used in this paper, a smoother deterioration characteristic curve is obtained.

**Figure 2:**
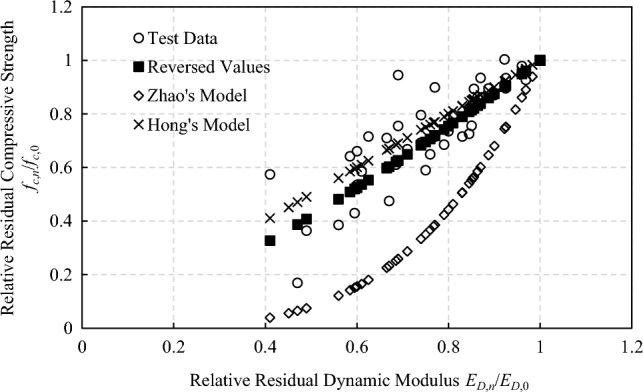
Reversed compressive strength of concrete under freeze–thaw action.

According to research results and test data in relevant literature^23–26^, the relative residual compressive strength of concrete under freeze–thaw action has a good correlation with the relative dynamic elastic modulus and follows the power function relationship. By fitting the data in the literature through power funtion, the relation between the residual compressive strength of concrete and the relative dynamic elastic modulus could be determined and shown in Fig. 2:

4 $$f_{c,n} /f_{c,0} = 0.99 \times (E_{D,n} /E_{D,0} )^{1.2796} + 0.01$$

where *f*_*c*,0_ is the initial compressive strength of concrete without freeze–thaw action; *f*_*c*,*n*_ is the compressive strength of concrete after *n* times of freeze–thaw action.

Zhao^26^ and Hong^6^ also proposed compressive strength deterioration models which are shown in Eqs. (5), (6) and Fig. 2.

5 $$f_{c,n} /f_{c,0} = (E_{D,n} /E_{D,0} \times 100)^{3.65} \times 5.01 \times 10^{ - 8}$$

6 $$f_{c,n} /f_{c,0} = E_{D,n} /E_{D,0}$$

It could be noted that the deterioration curve proposed in this paper lies in the middle of other models. Zhao's test data did not include cases where the relative residual dynamic elastic modulus is below 0.9 which makes the regressed deterioration curve much steeper when the relative residual dynamic elastic modulus decreases further. Hong thinks when damage degree is less than 40%, the compressive strength of freeze–thaw damaged concrete is not lower than 70% of its original strength. Therefore, the conservative estimate of the residual compressive strength of freeze–thaw damaged concrete is equivalent to the residual dynamic elastic modulus. As more experimental data are used in this paper, a relative compromised compressive strength deterioration characteristic curve is obtained.

Assuming that the proportional relationship between compressive strength and tensile strength of concrete under freeze–thaw action is the same as the standard value of concrete strength^27^, the tensile strength of concrete under freeze–thaw action could be determined as follows:

7 $$f_{c,n} {/}f_{t,n} = f_{ck} {/}f_{tk}$$

where *f*_*c*,*n*_, *f*_*t*,*n*_ are the compressive strength and tensile strength of concrete after *n* times of freeze–thaw action, respectively; *f*_*ck*_, *f*_*tk*_ are the standard compressive strength and tensile strength of concrete, respectively.

Equations (2)–(4) define respectively the static elastic modulus and strength deterioration process of concrete under freeze–thaw action.

## Reliability analysis method of arch dam

### Finite element equivalent stress method

An input–output model is needed to evaluate the reliability of arch dam structure. In the input–output model defined by finite element method, stress concentration could occur at the corner edge of the dam resulting in the decrease of the reliability index^28^. In order to reduce stress concentration, the finite element internal force analysis results will be treated by equivalent stress method in the post-processing stage^29^.

The stress at each node of arch dam *σ*_*ij*_′ in the global coordinate system (*x*′, *y*′, *z*′) is first calculated by finite element method. Then, a local coordinate system (*x*, *y*, *z*) with radial and tangential axes of the arch is established at node *k* of the horizontal arch circle, where the X-axis is parallel to the tangential direction of the arch center line, the Y-axis is parallel to the radius direction, and the Z-axis is the vertical direction. The coordinate’s origin is on the arch center line. The stress *σ*_*ij*_′ in the global coordinate system could be converted into the stress in the local coordinate system *σ*_*ij*_ = (*σ*_*x*_, *σ*_*y*_, *σ*_*z*_, *τ*_*xy*_, *τ*_*yz*_, *τ*_*zx*_)^*T*^ by means of the coordinate transformation matrix:

8 $$\left\{ \sigma \right\} = \left[ T \right]\left\{ {\sigma^{\prime } } \right\}$$

where [*T*] is the coordinate transformation matrix.

9 $$\left[ T \right] = \left[ {\begin{array}{*{20}c} {l_{1}^{2} } & {m_{1}^{2} } & {n_{1}^{2} } & {2l_{1} m_{1} } & {2m_{1} n_{1} } & {2l_{1} n_{1} } \\ {l_{2}^{2} } & {m_{2}^{2} } & {n_{2}^{2} } & {2l_{2} m_{2} } & {2m_{2} n_{2} } & {2l_{2} n_{2} } \\ {l_{3}^{2} } & {m_{3}^{2} } & {n_{3}^{2} } & {2l_{3} m_{3} } & {2m_{3} n_{3} } & {2l_{3} n_{3} } \\ {l_{1} l_{2} } & {m_{1} m_{2} } & {n_{1} n_{2} } & {l_{1} m_{2} + l_{2} m_{1} } & {m_{1} n_{2} + m_{2} n_{1} } & {l_{1} n_{2} + l_{2} n_{1} } \\ {l_{2} l_{3} } & {m_{2} m_{3} } & {n_{2} n_{3} } & {l_{2} m_{3} + l_{3} m_{2} } & {m_{2} n_{3} + m_{3} n_{2} } & {l_{2} n_{3} + l_{3} n_{2} } \\ {l_{1} l_{3} } & {m_{1} m_{3} } & {n_{1} n_{3} } & {l_{1} m_{3} + l_{3} m_{1} } & {m_{1} n_{3} + m_{3} n_{1} } & {l_{1} n_{3} + l_{3} n_{1} } \\ \end{array} } \right]$$

where (*l*_*i*_, *m*_*i*_, *n*_*i*_) is the direction cosine of coordinates *x*, *y* and *z*.

On the horizontal section of the beam, the unit width on the center line of the arch is taken. The width at point *y* could be 1 + *y*/*r*. *r* is the curvature radius of the arch center line. The internal force of the beam could be obtained by integrating the stress and its moment along the thickness direction:

10 $$W_{b} = - \int\limits_{ - T/2}^{T/2} {\sigma_{z} \left( {1 + \frac{y}{r}} \right)dy} \;({\text{Vertical}}\;{\text{force}})$$

11 $$M_{b} = - \int\limits_{ - T/2}^{T/2} {(y - y_{0} )\sigma_{z} \left( {1 + \frac{y}{r}} \right)dy} \;({\text{Bending}}\;{\text{moment}})$$

12 $$Q_{b} = - \int\limits_{ - T/2}^{T/2} {\tau_{zx} \left( {1 + \frac{y}{r}} \right)dy} \;({\text{Tangential}}\;{\text{shear}}\;{\text{force}})$$

13 $$V_{b} = - \int\limits_{ - T/2}^{T/2} {\tau_{zy} \left( {1 + \frac{y}{r}} \right)dy} \;({\text{Radial}}\;{\text{shear}}\;{\text{force}})$$

14 $$\overline{{M_{b} }} = - \int\limits_{ - T/2}^{T/2} {(y - y_{0} )\tau_{zx} \left( {1 + \frac{y}{r}} \right)dy} \;({\text{Torque}})$$

where *T* is the thickness of arch circle, *y*_0_ is the centroid coordinates of the beam section.

The radial section width of arch ring per unit height is defined as 1. The internal force of an arch could be obtained by integrating the arch stress and its moment along the thickness direction:

15 $$H_{a} = - \int\limits_{ - T/2}^{T/2} {\sigma_{x} dy} \;({\text{Horizontal}}\;{\text{thrust}})$$

16 $$M_{a} = - \int\limits_{ - T/2}^{T/2} {\sigma_{x} ydy} \;({\text{Bending}}\;{\text{moment}})$$

17 $$V_{a} = - \int\limits_{ - T/2}^{T/2} {\tau_{xy} dy} \;({\text{Radial}}\;{\text{shear}}\;{\text{force}}).$$

Since the shear stresses are paired, the vertical shear and torque of the arch could be determined without calculation. Assuming that *σ*_*z*_, *σ*_*x*_, *τ*_*zx*_ are distributed linearly from upstream to downstream, the stress of dam body could be calculated by the method of material mechanics according to the internal forces of arch and beam. The remaining stress components need to be obtained by taking the microelements from the upstream and downstream edges and solving the equilibrium equations of the microelements.

#### Downstream surface stress

The vertical normal stress of the cantilever beam on the horizontal plane could be calculated as follows:

18 $$\sigma_{zd} = \frac{{W_{b} }}{{A_{b} }} - \frac{{M_{b} }}{{I_{b} }}(t - \lg ).$$

The horizontal normal stress of the arch in the radial plane could be calculated as follows:

19 $$\sigma_{xd} = \frac{{H_{a} }}{{A_{a} }} - \frac{{M_{a} }}{{I_{a} }} \times \frac{t}{2}.$$

The tangential horizontal shear force of the horizontal cantilever beam could be calculated as follows:

20 $$\tau_{xzd} = \tau_{zxd} = - \frac{{Q_{b} }}{{A_{b} }} + \frac{{\overline{M}_{b} }}{{I_{b} }}(t - \lg ).$$

The other three stress components could be calculated as follows:

21 $$\tau_{xyd} = \tau_{yxd} = (\sigma_{xd} - P_{d} )\tan \eta_{d} - \tau_{xzd} \tan \varphi_{d}$$

22 $$\tau_{yzd} = \tau_{zyd} = (\sigma_{zd} - P_{d} )\tan \varphi_{d} - \tau_{xzd} \tan \eta_{d}$$

23 $$\sigma_{yd} = P_{d} + \tau_{xyd} \tan \eta_{d} + \tau_{yzd} \tan \varphi_{d}$$

where *φ*_*d*_ is the angle between the dam surface and the vertical direction in the radial vertical plane; *η*_*d*_ is the angle between the dam surface and the tangent line of the arch center line in the horizontal plane.

The three stresses parallel to the dam surface could be calculated as follows:

24 $$\sigma_{zd}^{\prime } = \sigma_{zd} \sec^{2} \varphi_{d}^{\prime } - P_{d} \tan^{2} \varphi_{d}^{\prime } = \sigma_{zd} (1 + \tan^{2} \varphi_{d}^{\prime } ) - P_{d} \tan^{2} \varphi_{d}^{\prime }$$

25 $$\begin{aligned} \sigma_{xd}^{\prime } & = \sigma_{xd} \cos^{2} \eta_{d} + \sigma_{yd} \sin^{2} \eta_{d} + 2\tau_{xyd} \sin \eta_{d} \cos \eta_{d} \\ & = (\sigma_{xd} + \sigma_{yd} \tan^{2} \eta_{d} + 2\tau_{xyd} \tan \eta_{d} )/(1 + \tan^{2} \eta_{d} ) \\ \end{aligned}$$

26 $$\tau_{xzd}^{\prime } = \tau_{zxd}^{\prime } = (\tau_{zxd} \cos \eta_{d} - \tau_{zyd} \sin \eta_{d} )\sec \varphi_{d}^{\prime } .$$

The equivalent principal stress of the downstream dam surface could be calculated as follows:

27 $$\sigma_{pd} = \left\{ {\begin{array}{*{20}l} {\frac{{\sigma_{zd}^{\prime } + \sigma_{xd}^{\prime } }}{2} + \sqrt {\left( {\frac{{\sigma_{zd}^{\prime } - \sigma_{xd}^{\prime } }}{2}} \right)^{2} + \left( {\tau_{xzd}^{\prime } } \right)^{2} } ,} \hfill & {\sigma_{zd}^{\prime } - \sigma_{xd}^{\prime } > 0} \hfill \\ {\frac{{\sigma_{zd}^{\prime } + \sigma_{xd}^{\prime } }}{2} - \sqrt {\left( {\frac{{\sigma_{zd}^{\prime } - \sigma_{xd}^{\prime } }}{2}} \right)^{2} + \left( {\tau_{xzd}^{\prime } } \right)^{2} } ,} \hfill & {\sigma_{zd}^{\prime } - \sigma_{xd}^{\prime } \le 0} \hfill \\ \end{array} } \right.$$

where

28 $$\tan \varphi_{d}^{\prime } = \tan \varphi_{d} \cos \eta_{d}$$

29 $$I_{b} = \frac{{R_{u} t^{3} }}{36r}\left[ {\frac{{1 + 4\frac{{R_{d} }}{{R_{u} }} + \left( {\frac{{R_{d} }}{{R_{u} }}} \right)^{2} }}{{1 + \frac{{R_{d} }}{{R_{u} }}}}} \right]$$

30 $$\lg = \frac{t}{3} \times \frac{{R_{u} + 2R_{d} }}{{R_{u} + R_{d} }}$$

31 $$I_{a} = \frac{{t^{3} }}{12}$$

where *t* is the arch ring thickness; *A*_*b*_ = *A*_*a*_ = *t*; *R*_*u*_ = *r* + *t*/2; *R*_*d*_ = *r* − *t*/2; *P*_*d*_ is the downstream dam surface pressure.

#### Upstream surface stress

The upstream dam surface stress calculation is the same as the downstream. The main calculation formulas are as follows:

32 $$\sigma_{zu} = \frac{{W_{b} }}{{A_{b} }} + \frac{{M_{b} }}{{I_{b} }} \times \lg$$

33 $$\sigma_{xu} = \frac{{H_{a} }}{{A_{a} }} + \frac{{M_{a} }}{{I_{a} }} \times \frac{t}{2}$$

34 $$\tau_{xzu} = \tau_{zxu} = - \frac{{Q_{b} }}{{A_{b} }} - \frac{{\overline{M}_{b} }}{{I_{b} }} \times \lg$$

35 $$\tau_{xyu} = \tau_{yxu} = - (\sigma_{xu} - P_{u} )\tan \eta_{u} - \tau_{xzu} \tan \varphi_{u}$$

36 $$\tau_{yzu} = \tau_{zyu} = - (\sigma_{zu} - P_{u} )\tan \varphi_{u} - \tau_{xzu} \tan \eta_{u}$$

37 $$\sigma_{yu} = P_{u} - \tau_{xyu} \tan \eta_{u} - \tau_{yzu} \tan \varphi_{u}$$

38 $$\sigma_{zu}^{\prime } = \sigma_{zu} \sec^{2} \varphi_{u}^{\prime } - P_{u} \tan^{2} \varphi_{u}^{\prime } = \sigma_{zu} (1 + \tan^{2} \varphi_{u}^{\prime } ) - P_{u} \tan^{2} \varphi_{u}^{\prime }$$

39 $$\begin{aligned} \sigma_{xu}^{\prime } & = \sigma_{xu} \cos^{2} \eta_{u} + \sigma_{yu} \sin^{2} \eta_{u} - 2\tau_{xyu} \sin \eta_{u} \cos \eta_{u} \\ & = (\sigma_{xu} + \sigma_{yu} \tan^{2} \eta_{u} - 2\tau_{xyu} \tan \eta_{u} )/(1 + \tan^{2} \eta_{u} ) \\ \end{aligned}$$

40 $$\tau_{xzu}^{\prime } = \tau_{zxu}^{\prime } = (\tau_{zxu} \cos \eta_{u} - \tau_{zyu} \sin \eta_{u} )\sec \varphi_{u}^{\prime }$$

41 $$\sigma_{pu} = \left\{ {\begin{array}{*{20}l} {\frac{{\sigma_{zu}^{\prime } + \sigma_{xu}^{\prime } }}{2} + \sqrt {\left( {\frac{{\sigma_{zu}^{\prime } - \sigma_{xu}^{\prime } }}{2}} \right)^{2} + \left( {\tau_{xzu}^{\prime } } \right)^{2} } ,} \hfill & {\sigma_{zu}^{\prime } - \sigma_{xu}^{\prime } > 0} \hfill \\ {\frac{{\sigma_{zu}^{\prime } + \sigma_{xu}^{\prime } }}{2} - \sqrt {\left( {\frac{{\sigma_{zu}^{\prime } - \sigma_{xu}^{\prime } }}{2}} \right)^{2} + \left( {\tau_{xzu}^{\prime } } \right)^{2} } ,} \hfill & {\sigma_{zu}^{\prime } - \sigma_{xu}^{\prime } \le 0} \hfill \\ \end{array} } \right.$$

42 $$\tan \varphi_{u}^{\prime } = \tan \varphi_{u} \cos \eta_{u}$$

where *P*_*u*_ is the upstream dam surface pressure.

### Response surface method

When the probability distribution of input variables is determined according to the test data or specification parameters, the response surface method could be adopted to analyze the structural reliability^30^. In response surface method, a fitted response surface is adopted to replace the complex functional equation, which is essentially an improvement of Monte Carlo method and could greatly improve the efficiency of reliability evaluation^31^. However, the calculation accuracy of response surface method depends on the fitting degree of the selected sample points near the limit state surface^32^. It is necessary to obtain sufficient samples near the limit state surface. But too much sampling will affect the computational efficiency, and has little effect on the accuracy improvement. Therefore, an improved sampling strategy and convergence criterion are proposed to achieve a balance between computational accuracy and efficiency.

The quadratic polynomial is usually selected as the response surface function form. Set *X* = (*x*_1_, *x*_2_, …, *x*_*n*_) as the random input variable. The response surface function without cross terms could be defined as follows:

43 $$Z = g(X) = a + \sum\limits_{i = 1}^{n} {b_{i} X_{i} + \sum\limits_{i = 1}^{n} {c_{i} X_{i}^{2} } } .$$

As the Latin hypercube sampling algorithm is selected for sampling, at least 2*n* + 1 samples will be needed to fit the response surface function containing *n* random variables. However, 2*n* + 1 samples may not be close enough to the limit state surface, so the fitting effect could not be determined. The optimal response surface could be determined by stepwise trial calculation, which is expressed as follows:

The *i*th step trial is defined to fit *k* response surfaces. The number of response sampling points used to fit each response surface is respectively *in* + *n* + 1, (*i* + 1)*n* + *n* + 1, (*i* + 2)*n* + *n* + 1, …, (*i* + *k*-1)*n* + *n* + 1. The target probability of each response face is solved separately as *P*_*f,i*_, *P*_*f,i*+1_, *P*_*f,i*+2_, *…*, *P*_*f,i*+*k*-1_. If the following formula is true:

44 $$\left[ {\max \left( {P_{f} } \right) - \min \left( {P_{f} } \right)} \right]/{\text{E}}\left( {P_{f} } \right) < \varepsilon$$

where max(*P*_*f*_), min(*P*_*f*_), E(*P*_*f*_) are respectively the maximum value, minimum value and mean value of the target probability in the set of stepped response surfaces, *ε* is the allowable error.

Then, it is considered that the *i*th step trial calculation has achieved a suitable fitting effect near the limit state surface. The response surface whose target probability is closest to E(*P*_*f*_) will be taken as the representative fitting response surface. Otherwise, *i* + 1 step trial calculation will be conducted, and *n* sample points will be newly added on the basis of the original sample points for trial test until Formula (41) is satisfied.

In this method, the number of sample points is gradually expanded, and the fitting effect is constantly self-evaluated so as to determine the best sample point number and the corresponding response surface. The essence is to propose a computational convergence criterion.

## Case study

### Project overview

A concrete arch dam located in the northwest of China is studied. The maximum height is 81.6 m. The top elevation is 401.60 m, the bottom elevation is 320.00 m, the top width is 5.0 m, and the bottom width of the crown cantilever is 15.0 m. The dam was constructed amd completed in 1973 and has been in service for 50 years. The shape parameters of the arch dam are shown in Table 1.

**Table 1:** Shape parameters of the arch dam.

Elevation *Z* (m)	Exceeding value △*x* (m)	Arch thickness *T* (m)	Center line radius of arch ring *R* (m)	Center angle of arch ring		
				Left bank	Right bank	Total
401.60	0.000	5.000	124.500	54° 00′	54° 00′	108° 00′
397.00	0.000	5.000	124.500	52° 00′	52° 00′	104° 00′
386.00	3.398	6.429	118.786	51° 00′	51° 00′	102° 00′
375.00	5.878	7.857	113.571	49° 00′	49° 00′	98° 00′
364.00	7.439	9.286	107.357	46° 00′	46° 00′	92° 00′
353.00	8.082	10.714	99.643	42° 00′	42° 00′	84° 00′
342.00	7.806	12.143	89.429	37° 00′	38° 00′	75° 00′
331.00	6.612	13.571	80.714	32° 00′	33° 00′	65° 00′
320.00	4.500	15.000	72.500	28° 00′	28° 00′	56° 00′

The normal water level of the reservoir is 398.00 m, and the corresponding downstream water level is 341.70 m. The check flood level is 399.80 m, and the corresponding downstream water level is 343.00 m. The dead water level is 351.00 m, and the corresponding downstream water level is 326.50 m. The elevation of silt deposited in front of the dam is 328.00 m.

**Figure 3:**
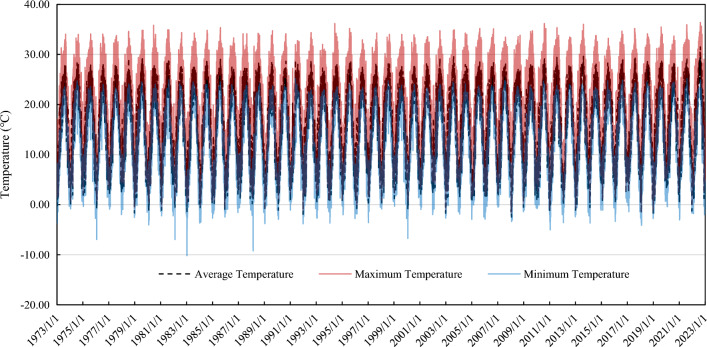
Temperature change process at the dam site.

The temperature change process of the dam site is shown in Fig. 3. The average annual temperature at the dam site is 15.9 °C with an annual temperature amplitude of 16 °C.

### Finite element model

**Figure 4:**
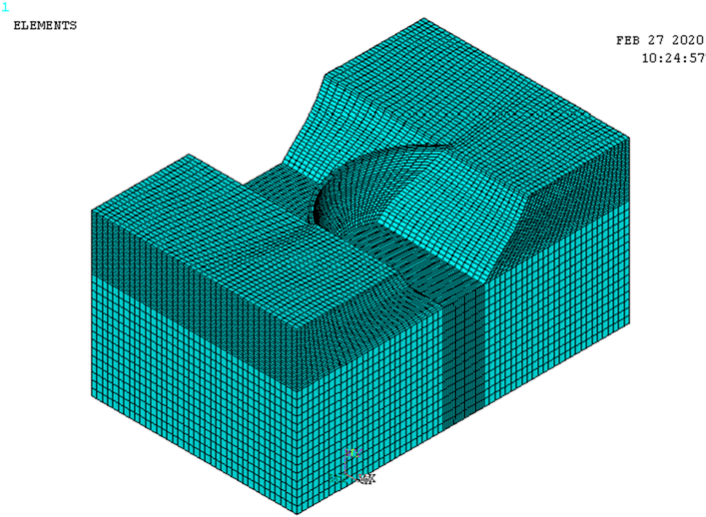
Model overall mesh.

**Figure 5:**
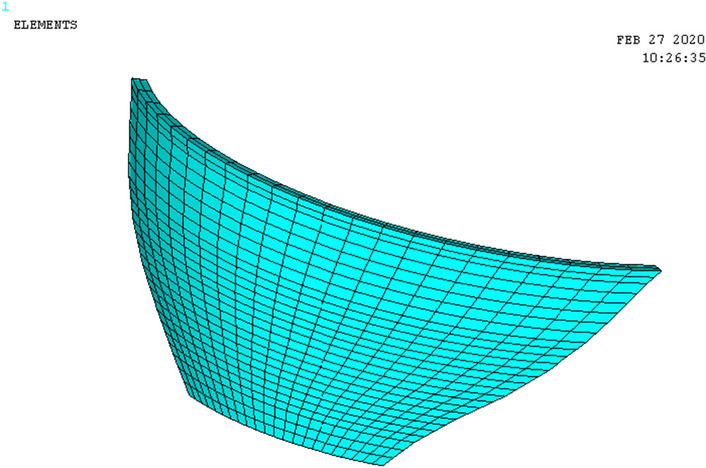
Arch dam body mesh.

The bedrock is argillaceous limestone which is hard and solid. Thus, the linear elastic model and hexahedral eight-node entity units are adopted to simulate the dam body and foundation. The calculation model is discretized into 101,228 nodes and 92,805 cells. The dam foundation model extends 2.5 times the length from upstream to downstream, 1.5 times the width from left bank to right bank of the dam abutment, and 2 times the arch height downward from the bottom of the arch dam (coordinate system: the origin is located at the vertex of the upstream surface of the arch beam, the X-axis is along the water flow direction, the upstream direction is negative, the downstream direction is positive, the Y-axis is along the vertical direction, the upward is positive, the downward is negative, the Z-axis is from right bank to left bank, the left bank is positive, the right bank is negative). Rigid constraints are applied to the bottom surface of the foundation, and linkage constraints are applied on the lateral surface along the direction of water flow. The overall model mesh is shown in Fig. 4. The arch dam body mesh is shown in Fig. 5.

### Working conditions, loads and parameters

According to the actual operation of the arch dam and “Design specification for concrete arch dams (SL282-2018)”^33^, the working conditions of the dam strength reliability analysis could be determined and shown in Table 2.

**Table 2:** Dam structural reliability calculation conditions.

Serial number	Working conditions	Loads					
		Gravity load	Water weight	Hydrostatic pressure	Uplift pressure	Sediment pressure	Temperature load
(1)	Normal water level and temperature drop	√	√	√	√	√	√
(2)	Normal water level and temperature rise	√	√	√	√	√	√
(3)	Dead water level and temperature drop	√	√	√	√	√	√
(4)	Dead water level and temperature rise	√	√	√	√	√	√
(5)	Check flood level and temperature rise	√	√	√	√	√	√

Temperature load is one of the main loads of arch dam^34^. Recent studies show that the arch sealing temperature and residual temperature are the main sources of temperature load uncertainty^35^. In this paper, the arch sealing temperatures are treated as random variables to characterize the uncertainty of temperature load. The arch sealing temperatures of eight layers of arch rings from arch bottom to arch top were separately considered as independent random variables. The coefficient of variation is set as 0.05. Temperature load is calculated according to “Design specification for concrete arch dams (SL282-2018)”^33^ considering average body temperature *T*_*m*_ and equivalent linearly-distributed temperature difference *T*_*d*_. The temperature load when the arch sealing temperature is 16.0 °C is shown in Table 3. The thermal linear expansion coefficient is taken as 8 × 10^−6^.

**Table 3:** Temperature load with the arch sealing temperature of 16.0 °C.

Serial number	Temperature load	Elevation (m)								
		401.60	397.00	386.00	375.00	364.00	353.00	342.00	331.00	320.00
(1)	*T*_*m*_	− 13.37	− 13.37	− 9.44	− 7.83	− 7.15	− 7.02	− 7.15	− 4.85	− 5.30
	*T*_*d*_	0.00	0.00	− 4.85	− 6.70	− 7.22	− 6.70	− 5.52	1.47	2.05
(2)	*T*_*m*_	13.17	13.17	10.04	5.89	3.05	1.10	− 0.32	− 1.74	− 3.84
	*T*_*d*_	0.00	0.00	3.25	10.18	15.01	18.14	20.06	14.82	9.13
(3)	*T*_*m*_	− 13.37	− 13.37	− 12.87	− 11.49	− 9.79	− 8.27	− 4.64	− 4.37	− 2.83
	*T*_*d*_	0.00	0.00	0.00	0.00	0.00	0.00	− 5.87	− 7.41	− 1.20
(4)	*T*_*m*_	13.17	13.17	12.67	11.29	9.59	8.07	5.75	2.86	1.07
	*T*_*d*_	0.00	0.00	0.00	0.00	0.00	0.00	3.25	10.03	12.19
(5)	*T*_*m*_	13.17	14.12	9.13	5.34	2.72	0.87	1.12	− 2.01	− 4.03
	*T*_*d*_	0.00	− 3.31	5.11	11.49	15.88	18.72	22.41	14.04	8.62

According to laboratory test, the elastic modulus of dam body varies from 16.8 to 22.6GPa. The standard deviation is 2.28GPa. Thus, the median value of 19GPa is adopted and variation coefficient is taken as 2.28/19 = 0.12. Other material property parameters are generally taken in the same way. The Initial tensile strength of concrete defined according to Eq. (4).

The dam body is made of C15 concrete. According to the laboratory test, the recommended initial elastic modulus is 19GPa. The Poisson's ratio is 0.20. The density is 2350 kg/m^3^. The initial tensile strength is 2.74 MPa.

The foundation is argillaceous limestone. According to the laboratory test, the recommended elastic modulus is 6 GPa. The Poisson's ratio is 0.25. The density is 2600 kg/m^3^.

The arch sealing temperature is usually taken as the temperature that is a little higher than the average annual temperature at the dam site, which is 15.9 °C. Thus, the arch sealing temperature is taken as 16.0 °C.

Random variables and their statistic parameters are shown in Table 4.

**Table 4:** Random variables and statistic parameters.

Random variables	Mean value	Variation coefficient	Distribution pattern
Upstream water depth (m)	78/31/79.8	0.01	Normal
Downstream water depth (m)	21.7/6.5/23	0.02	Normal
Initial elastic modulus of dam body *E*_*S,*0_ (GPa)	19	0.12	Normal
Dam body density (kg/m^3^)	2350	0.02	Normal
Elastic modulus of dam foundation (GPa)	6	0.12	Normal
Dam foundation density (kg/m^3^)	2600	0.02	Normal
Initial compressive strength of concrete *f*_*c,*0_ (MPa)	35.5	0.05	Normal
Initial tensile strength of concrete* f*_*t,*0_ (MPa)	2.74	0.05	Normal
Arch sealing temperature (°C)	16.0	0.05	Normal

The water depth corresponds to the normal storage level, dead level and check flood level respectively.

Because the compressive strength of arch dam is generally rich, and arch dams are mostly damaged by tension, the failure mode is selected as tensile failure in the structural reliability analysis.

### Results and discussions

**Figure 6:**
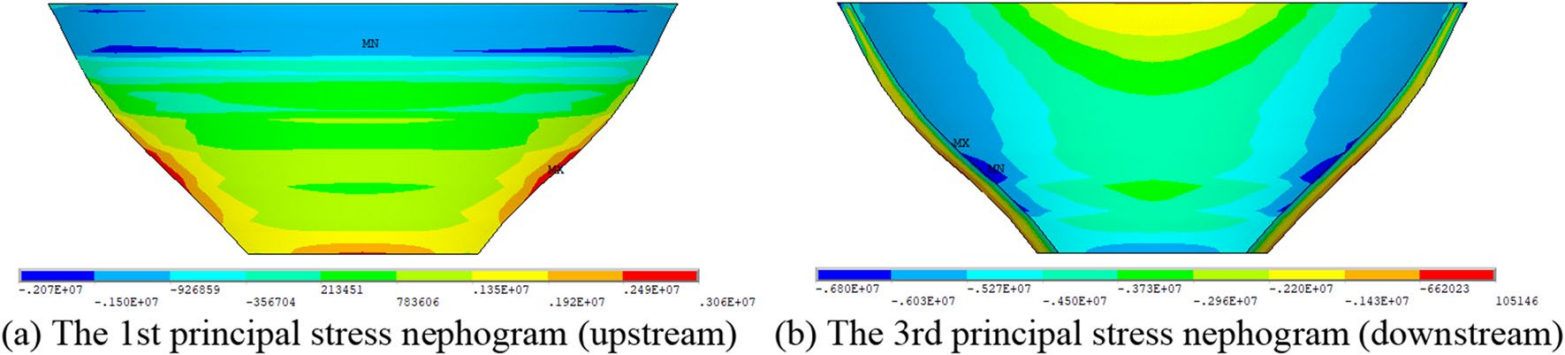
Principal stress nephogram of working condition 2.

The initial mean value of each parameter is adopted to simulate the initial dam stress state. As shown in Fig. 6 (the finite element stress is positive in tensile stress and negative in compressive stress), the maximum principal tensile stress is 3.06 MPa and the maximum principal compressive stress is 6.80 MPa at the basic load combination condition, both of which occur in the normal water level and temperature rise condition (Working Condition 2). As the occurrence frequency of working condition (2) is much higher than other working conditions, this working condition becomes the actual control condition of arch dam reliability.

The nephogram of principal stress shows that the finite element simulation produces local stress concentration. Thus, equivalent stress treatment is adopted and the results are shown in Table 5. The stress concentration is alleviated after treatment.

**Table 5:** Finite element equivalent stress results of working condition (2), unit: MPa.

Working condition	Upstream maximum principal stress		Downstream maximum principal stress	
	Tensile stress	Compressive stress	Tensile stress	Compressive stress
(1)	1.56	4.35	1.19	4.52
(2)	1.96	2.95	0.78	5.20
(3)	1.28	2.36	1.98	2.11
(4)	0.82	1.61	1.08	3.72
(5)	2.21	3.78	1.05	5.31

The maximum stress locations are basically the same as the location of direct finite element calculation results.

The response surface method proposed in this paper is adopted to evaluate the structural reliability. According to the definition of aggregation number *k* of stepped response surfaces expressed above, the larger the aggregation number *k* is, the harsher the convergence condition will be. Generally, the calculation results will be more secure, meanwhile the corresponding amount of calculation will be increased. In fact, a good convergence could be achieved when *k* ≥ 3. Considering the calculation accuracy and efficiency comprehensively, the aggregation number of stepped response surfaces *k* is set to be 4, and the allowable error *ε* is set to be 0.5.

**Figure 7:**
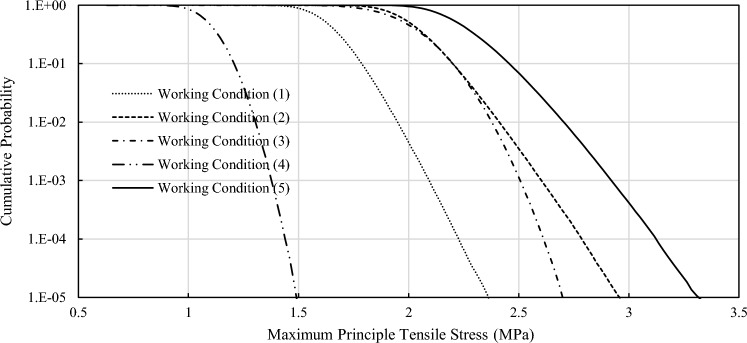
Cumulative probability curve of maximum principal tensile stress under initial condition.

In order to directly reflect the cumulative probability distribution of structural stress, the maximum principal tensile stress is taken as the objective function. The initial concrete tensile strength is taken as a constant to solve the structural failure probability at initial state (without freeze–thaw effect). The stepwise response surface convergence process is shown in Table 6. The failure probability reaches relative stability in step 5, and the calculation effect has been guaranteed with 121 samples. According to the response surface selection strategy described above, response surface 7 is selected. The cumulative probability curve of maximum principal tensile stress output by this response surface is shown in Fig. 7.

**Table 6:**
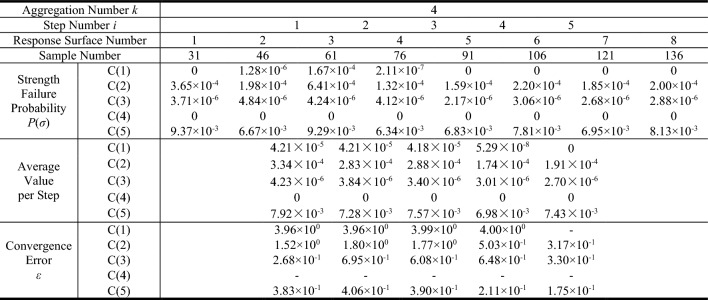
Failure probability convergence process through response surface method.

C(*) refers to working condition (*).

Considering the annual occurrence of each working condition as mutually independent events, the annual structural failure probability could be estimated by the following formula:

45 $$P_{f} \approx 1 - \prod\limits_{i = 1}^{5} {[1 - P(\sigma )_{i} P(C)_{i} ]}$$

where *P*(*σ*)_*i*_ is the strength failure probability of each working condition, *P*(*C*)_*i*_ is the annual occurrence probability of each working condition which could be replaced by statistical frequency.

**Figure 8:**
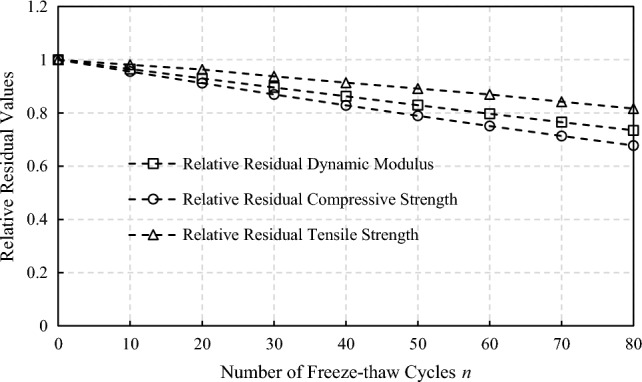
Time-varying process of the dam material properties under freeze–thaw action (mean value).

**Figure 9:**
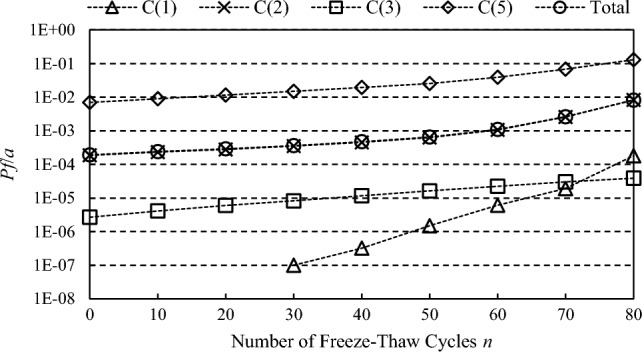
Time-varying process of the arch dam failure probability under freeze–thaw action.

It is assumed that a freeze–thaw cycle is completed when the daily minimum temperature is below − 1 °C for over five consecutive days followed by five consecutive days above -1 °C. According to the statistics of the reservoir operating temperature shown in Fig. 3, 29 freeze-thawing times have occurred so far. According to the freeze–thaw deterioration model of concrete proposed in this paper, the time-varying process of the dam material properties and arch dam failure probability under freeze–thaw action are calculated, as shown in Figs. 8 and 9.

According to the calculation results, it could be known that:

1. Compared with the dead water level working condition, the maximum principal tensile stress cumulative probability curve in the condition with higher water level ((1), (2), (5)) has lower slope and longer span, which indicates that the possible stress fluctuation range of arch dam body is larger and the arch structure is in a relatively unstable state under high water pressure load.
2. The calculation results of normal water level & temperature rise condition (working condition (2)) are close to that of dead water level & temperature drop condition. However, since the former happens almost every year after impoundment, it is the actual control condition of the failure probability of arch dam. The other working conditions occur less frequently in one year and have little influence on the annual failure probability. Therefore, the failure probability of arch dam is approximately equal to the failure probability of normal water level & temperature rise condition (working condition (2)).
3. With the increase of freeze-thawing times, the failure probability of arch dam structure generally increases and the reliability decreases. At early stage of freeze–thaw action (approximately within 50 times of freeze–thaw cycles), though the tensile strength of concrete is decreasing, the failure probability will increase steadily in a small range, and the reliability of the arch dam structure will not decrease significantly. Because the stress level of dam body is also relieved when the elastic modulus is reduced. After that, as the tensile strength of concrete continue to decrease, the structural failure probability will suddenly increase. After 80 times of freeze-thawing cycles, the annual failure probability of the arch dam structure will reach about 1% and the arch dam structure will be unreliable.
4. Although the residual strength decreases slowly due to freeze–thaw effect, it has a great influence on the reliability of the structural strength. Because the residual tensile strength slowly approaches the maximum tensile stress of the structure. As the dam has freeze–thaw action 29 times during its 50 years of service, it is estimated that the dam will reach a 1% probability of failure in about 88 years. The problem of freeze–thaw damage should be paid attention to and corresponding pre-strengthening measures should be taken.

## Conclusions

The material of concrete arch dam is easy to deteriorate under freeze–thaw action which affects its structural reliability and service life. In this paper, a freeze–thaw deterioration model of mechanical properties of concrete materials is established based on measured data. A response surface method with improved convergence criterion is proposed. The time-varying reliability process of arch dam structure under freeze–thaw action is studied. The main conclusions could be made as follows:

1. The relationships between freeze–thaw cycles, concrete dynamic elastic modulus, static elastic modulus and compressive and tensile strength are analyzed. A freeze–thaw deterioration model of concrete is established through regression analysis to simulate the deterioration process of mechanical properties which could provide a simple and practical analysis method for engineering application.
2. In view of the contradiction between the computation effect and computation amount of response surface method, a convergence criterion and strategy for sampling is proposed so as to determine the relatively optimal response surface. The case study results show that the response surface fitted after the fifth step trial is basically stable, and the fluctuation is not obvious. To some extent, the proposed convergence criterion could guarantee the effectiveness of response surface calculation, avoid excessive sampling quantity, and reduce the amount of calculation. It is feasible to use the convergence criterion proposed in this paper to determine the relative optimal response surface.
3. The simulation results of time varying structural reliability of arch dam show that, at early stage of freeze–thaw, though the tensile strength of concrete is decreasing, the reliability of the arch dam structure will not decrease significantly, because the stress level of dam body is also relieved when the elastic modulus is reduced. However, as the tensile strength continues to decrease under freeze–thaw action, the failure probability will increase rapidly. After 50 times of freeze-thawing cycles, the structural reliability will suddenly fall off a cliff. As the dam has freeze–thaw action 29 times during its 50 years of service, it is estimated that the dam will reach a 1% probability of failure in about 88 years. The problem of freeze–thaw damage should be paid attention to and corresponding pre-strengthening measures should be taken. Learning from the stress distribution, the stress level of the arch end of both sides is high, and the weak parts such as the arch end of both sides are suggested being monitored and maintained in daily operation.

## Data Availability

All associated data have been presented in the manucript which are available from the corresponding author on reasonable request.
